# Philadelphia Dispensary

**Published:** 1839-04-13

**Authors:** 


### PHILADELPHIA DISPENSARY.

*Report for March*, 1839.

The whole number of persons who have received medical aid from the institution, during the month, is 235,—of whom 126 were prescribed for at the dispensary, and 109 at their own dwellings.

Of the latter, 44 were males, and 65 females,—87 natives, and 22 foreigners,—97 white, and 12 coloured,—99 were cured, and 10 died.

The deaths were from pneumonia, phthisis, and meningitis, each two,—from chronic bronchitis, pertussis, scarlatina, and scrofula, each one.

The diseases of most frequent occurrence were catarrh, pneumonia, and rheumatism.

In addition to the above cases, several were sent to the almshouse or hospital during their treatment, and none yet remaining are included in the report.

